# A hybrid filtering method with no-reference quality assessment for synthetic aperture sonar images

**DOI:** 10.1371/journal.pone.0332458

**Published:** 2025-11-18

**Authors:** Zhiping Xu, Deyin Xu, Yisong He, Lixiong Lin, Jiachun Zheng

**Affiliations:** 1 School of Ocean Information Engineering, Jimei University, Xiamen, China; 2 Key Laboratory of Underwater Acoustic Communication and Marine Information Technology (Xiamen University), Ministry Education, Xiamen, China; Whale Wave Technology Inc., CHINA

## Abstract

Synthetic Aperture Sonar (SAS) imaging technology is wildly used in the underwater applications. In the work process of SAS imaging, filtering technologies are important for SAS imaging, which can suppress different noises to improve signal quality. However, the existing filtering methods face many challenges, such as insufficient noise suppression, degradation of image detail, edge blurring and so on. Furthermore, the existing quality assessments for filtering methods are sometimes subjective, which limits the research development for filtering technologies. To solve these problems, we propose a hybrid filtering method with a no-reference quality assessment for SAS images in this paper. The proposed method includes two-stages, the first stage is to suppress local statistical interference, and the second stage is to preserve edge information by weighted smoothing. With the no-reference quality assessment, the hybrid filtering method and other filtering methods, including mid-value filtering and mean-value filtering methods, are investigated. The numerical results show that the no-reference quality assessment method can efficiently analyze different filtering methods, and the proposed methods can perform better than other filtering methods.

## 1 Introduction

In underwater environments where traditional optical and electromagnetic sensing methods are limited, severe challenges arise in human cognition of the underwater world due to light attenuation and the short propagation distance of electromagnetic waves. Consequently, underwater regions are often referred to as “blind spots" in human perception [1,2]. Synthetic aperture sonar (SAS) imaging, as an acoustic wave-based imaging technique, effectively overcomes these limitations by leveraging the physical property that sound waves propagate efficiently and over long distances in water [3]. Currently, sonar imaging has been widely applied in key fields such as fundamental scientific research, national security, engineering construction, and resource exploration, becoming a “core infrastructure" supporting the development of intelligent underwater perception systems. With continuous improvements in resolution and imaging accuracy, sonar images are steadily expanding the boundaries of human perception of the deep-sea world [4–7].

In the SAS image processing workflow, filtering technology plays a crucial role as a key preprocessing step [8,9]. Its primary objectives are to suppress multi-source noise and enhance target signals, thereby providing a clear and reliable image foundation for subsequent high-level tasks such as target detection, terrain mapping, and ecological analysis [10–12]. The noise components in SAS images are complex, commonly including random noise, stripe noise, impulse noise, and attenuation or blurring caused by the propagation process. These factors directly affect the usability and analytical accuracy of images, for example, increasing false detection rates or causing inaccurate terrain boundaries. Therefore, filtering is regarded as the “first line of defense” in the SAS image analysis process [13–17].

Through proper filtering operations, noise interference can be effectively suppressed, significantly enhancing image quality [18–20]. For example, after noise suppression, previously obscured target details become visible, and edge structures in the image become clearer, which helps improve the accuracy of subsequent recognition and analysis [21,22]. Furthermore, image enhancement filtering techniques can adjust image contrast and brightness, highlighting edges and texture features, thereby assisting operators in intuitively judging spatial characteristics of targets such as position, shape, and size [23–26]. Additionally, filtering can emphasize key features in the image (such as texture, edges, and geometric shapes) and strengthen the contrast between targets and background, thereby significantly improving target recognition accuracy and reliability, ultimately providing more valuable visual information for underwater operations [12,27]. Among numerous filtering methods, median filtering and mean filtering are widely used due to their simplicity and effectiveness. Median filtering, proposed by J.W. Tukey in 1971, is a nonlinear filtering method based on order statistics theory, which effectively removes impulse noise while smoothing the image and preserving edge information. However, classical median filters may cause loss of image detail during noise reduction [28]. To address this issue, various improved algorithms have been proposed. For instance, Brownrigg introduced weighted median filtering, which assigns different weights to pixels within the filtering window to prioritize noise suppression and protect signal points. Additionally, variants based on fuzzy logic and adaptive mechanisms have emerged to further balance noise removal capability and detail preservation [29–31]. Signal processing challenges in underwater environments extend beyond sonar imaging to encompass various sensor modalities used in marine robotics and autonomous underwater vehicles (AUVs). Similar to SAS imaging, these systems often employ rotary encoders and position sensors that face comparable noise interference and signal degradation issues [32,33]. The fundamental principles of noise suppression and signal enhancement developed for such applications can provide valuable insights for improving sonar image processing methodologies.

In contrast, mean filtering [34], as a linear image smoothing technique, works by averaging pixel values within a local neighborhood to reduce local grayscale variations, thereby suppressing random noise in the image. Since the 1960s, mean filtering has been extensively applied in fields such as medical image processing, remote sensing image analysis, and computer vision [35]. Although this method performs well in noise suppression, its ability to protect image details is limited. With advancements in image processing algorithms and computational resources, modern research is attempting to integrate traditional mean filtering methods with new technologies such as deep learning and adaptive control to enhance performance and intelligence across diverse application scenarios [36]. In the process of SAS image processing and filter algorithm optimization, image quality evaluation methods play an indispensable role [37,38]. Acting as a bridge between theoretical algorithms and practical applications, quality evaluation methods not only provide objective measurements of filtering effects but also serve as the foundation for technological iteration, optimization, and standardization. By clearly defining evaluation metrics such as signal-to-noise ratio (SNR), mean squared error (MSE), and structural similarity index (SSIM), the filtering performance can be comprehensively assessed in terms of noise suppression, detail preservation, and edge integrity. These metrics offer unified quantitative standards for comparing different filtering algorithms or parameter settings and provide guidance for algorithm selection and system tuning [39–42].

Moreover, combining image quality evaluation metrics with algorithm performance indicators (such as computational complexity, runtime, and memory consumption) helps find an optimal balance between effectiveness and resource usage, improving overall system efficiency and practicality [43–45]. By analyzing trends in evaluation metrics, researchers can identify current algorithm limitations and further promote developments toward “stronger noise suppression, less detail loss, and higher adaptability.” Meanwhile, a unified quality evaluation system facilitates the establishment of industry standards, enhancing comparability and reproducibility of results across different research teams and application platforms, thus avoiding discrepancies caused by inconsistent standards [46–49].

In summary, considering the importance of SAS imaging, filtering as a fundamental preprocessing technology undertakes the dual responsibilities of noise suppression and information enhancement, while image quality evaluation methods provide scientific and quantitative assessment criteria. Together, these three elements form a tightly integrated technical loop within intelligent underwater perception systems, serving as key driving forces for continuous advancement in sonar image processing technology and the deep integration of theory and practice [50–52].

In this paper, we propose a two-stage hybrid filtering method, including local statistical interference suppression and edge-preserving weighted smoothing, to solve the problems in SAS imaging. The no-reference quality assessment is also adopted to evaluate the performance of the filtering methods. The experimental results show the proposed filtering method can perform better than other filtering methods with good filtering effect.

## 2 Methodology

### 2.1 Hybrid filtering

To enhance the processability and robustness of the original image in subsequent analysis tasks, this paper designs and adopts a two-stage image preprocessing framework, which respectively achieves robust suppression of local strong interference and edge-sensitive nonlinear smoothing reconstruction. The algorithm is described as follows.

**Stage One: Local statistical interference suppression**

Let the input image be $I:\Omega \to {\mathbb{R}}^{3}$, where $\Omega \subset {\mathbb{Z}}^{2}$ is the 2D spatial domain, and $I(x,y)=[I_{R}(x,y),I_{G}(x,y),I_{B}(x,y){]}^{⊤}$ is the RGB intensity vector at pixel (*x*,*y*), with $I_{R}(x,y),I_{G}(x,y),I_{B}(x,y)$ denoting the red, green, and blue intensity components, respectively. To address isolated extreme pixel values that may appear in the image, this paper first performs local statistical mapping on each color channel c∈{R,G,B} individually. Specifically, for any pixel location (*x*,*y*), its local neighborhood is defined as *N*_*k*_(*x*,*y*), where k×k denotes the neighborhood size, and the local intensity set is constructed as:

$$S_{c}(x,y)=\{I_{c}(i,j)|(i,j)\in N_{k}(x,y)\}$$ (1)

$$N_{k}(x,y)=\{(i,j)\in \Omega ||i-x|\leq r,|j-y|\leq r,\;k=2r+1\}$$ (2)

Based on this, a sorting statistical operation is applied to the set *S*_*c*_(*x*,*y*), from which a representative central statistic ${\hat{I}}_{c}(x,y)$ is extracted as the updated value for the current pixel, generating the intermediate image $I_{c}^{\prime }(x,y)$. The specific calculation is as follows:

$$S_{c\_sorted}=[v_{0},v_{1},\cdots ,v_{k-1}],\;v_{0}\leq v_{1}\leq \cdots \leq v_{k-1}$$ (3)

where $S_{c\_sorted}$ denotes the sorted version of *S*_*c*_(*x*,*y*), and $v_{k-1}$ represents the maximum pixel intensity in the neighborhood;

$${\hat{I}}_{c}(x,y)=\left\{ \begin{array}{cc} v_{(k-1)/2}, & \text{if }k\text{isodd}\\ \frac{v_{k/2-1}+v_{k/2}}{2}, & \text{if }k\text{ is even} \end{array}\right.$$ (4)

**Stage Two: Edge-preserving weighted smoothing**

For each pixel location (*x*,*y*) in channel *c*, its smoothed output value ${I_{c}^{\prime }}^{\prime }(x,y)$ is defined as:

$${I_{c}^{\prime }}^{\prime }(x,y)=\frac{1}{Z_{c}(x,y)}\sum_{\left(i,j)\in N_{d}(x,y\right)}I_{c}^{\prime }(i,j)\cdot \exp\left(-\frac{(I_{c}^{\prime }(i,j)-I_{c}^{\prime }(x,y){)}^{2}}{2\sigma_{r}^{2}}-\frac{||(i,j)-(x,y)|{|}^{2}}{2\sigma_{s}^{2}}\right)$$ (5)

where *N*_*d*_(*x*,*y*) denotes the spatial neighborhood centered at pixel (*x*,*y*), exp(·) is the exponential function, $||\;\cdot \;|{|}^{2}$is the squared Euclidean distance between two 2D coordinates. $\sigma_{r}>0$ is the standard deviation in the intensity domain, used to measure pixel value similarity and control the influence of color consistency on the weights; $\sigma_{s}>0$ is the standard deviation in the spatial domain, used to measure geometric distance and suppress the influence of distant regions on the current pixel. *Z*_*c*_(*x*,*y*) is the normalization factor, defined as:

$$Z_{c}(x,y)=\sum_{\left(i,j)\in N_{d}(x,y\right)}\exp\left(-\frac{(I_{c}^{\prime }(i,j)-I_{c}^{\prime }(x,y){)}^{2}}{2\sigma_{r}^{2}}-\frac{||(i,j)-(x,y)|{|}^{2}}{2\sigma_{s}^{2}}\right)$$ (6)

This weighting mechanism constructs an adaptive local fusion function centered on intensity similarity and spatial proximity. Its core advantage lies in performing significant smoothing in structurally consistent regions while preserving edges from being blurred, thereby significantly improving visual consistency and structural fidelity of the image.

In summary, the first-stage operation has nonlinear and non-local mean characteristics, which can significantly reduce the impact of impulse-like disturbances and avoid edge blurring caused by linear smoothing. While the first stage effectively mitigates extreme pixel interference, low-amplitude high-frequency noise may still exist in real images, especially in textured areas. The second stage adopts a structure-aware nonlinear filtering method that accounts for both spatial proximity and intensity similarity to reconstruct the result of the first stage with detail preservation and noise suppression.

### 2.2 No-reference quality assessment method

A method, named Unified Quality Assessment method for Sonar Imaging and Processing (UASIP), was proposed in [50], whose core concept is *Attribute Consistency*. Specifically, the UASIP method evaluates image quality from both perceptual and structural perspectives, and defines four key quality attributes:

- Region Discrimination (RD): assesses the separability between targets and background in the grayscale domain.
- Geometric Integrity (GI): reflects the fidelity of the target’s spatial structure.
- Detail Preservation (DP): evaluates the richness and spatial distribution of texture information.
- Cleanness Level (CL): measures the degree of noise suppression and background interference.

Based on these attributes, the method constructs a lightweight 10-dimensional feature vector that comprehensively represents the above information. This vector is then mapped to a continuous quality score using Support Vector Regression (SVR), enabling effective and objective sonar image quality assessment.

#### 2.2.1 Region discrimination.

Global Region Discrimination is quantified by the Kullback-Leibler (KL) divergence between the grayscale histogram *f*(*t*) of the image and a uniform distribution *g*(*t*):

$$D_{\text{KL}}(f∥g)=\sum_{t=0}^{255}f(t)\log\left(\frac{f(t)}{g(t)}\right)$$ (7)

Local Region Discrimination is modeled using local contrast maps:

$$L(x,y)=\sigma_{L}(x,y{)}^{\lambda },\;\lambda =3.75,$$ (8)

where $\sigma_{L}(x,y)$ denotes the local standard deviation. Robust features are then extracted using the diagonal wavelet coefficients *W*_*d*1_:

$$f_{\text{LD}}=\log_{10}\left(W_{d1}+\left|\min(W_{d1})\right|\right)$$ (9)

#### 2.2.2 Geometric integrity.

Geometric fidelity is evaluated via contour detection under a biologically-inspired inhibition mechanism. A positive response image is first generated using a Difference of Gaussian (DoG) operation. After applying a Heaviside function H(·), a normalized weight map is obtained:

$$W_{s}(x,y)=\frac{H({\text{DoG}}_{s}^{+}(x,y))}{\|H({\text{DoG}}_{s}^{+}(x,y)){\|}_{1}}$$ (10)

This is combined with the edge mask *M*_*s*_(*x*,*y*) to compute the suppressed contour response:

$$S(x,y)=M_{s}(x,y)\cdot W_{s}(x,y)$$ (11)

A Generalized Gaussian Distribution (GGD) is then fitted to *S*(*x*,*y*):

$$p(x;\alpha ,\sigma^{2})=\frac{\alpha }{2\beta \;\tau (1/\alpha {)}^{\alpha }}\exp\left(-{\left(\frac{\left|x\right|}{\beta }\right)}^{\alpha }\right)$$ (12)

with:

$$\beta =\sigma \sqrt{\frac{\tau (1/\alpha )}{\tau (3/\alpha )}}$$ (13)

Additionally, the entropy of the contour image is calculated to quantify geometric complexity:

$$S_{\text{Ent}}=-\sum_{k=0}^{255}p_{k}\log_{2}p_{k}$$ (14)

#### 2.2.3 Detail preservation.

Detail preservation is modeled using the Gray-Level Co-occurrence Matrix (GLCM):

$$G(i,j;d,\theta )=\sum_{x=0}^{W-1}\sum_{y=0}^{H-1}\varepsilon_{ij}\left(I(x,y),I(x+d\cos\theta ,y+d\sin\theta )\right)$$ (15)

where *d* = 1 and $\theta \in \{0^{\circ },45^{\circ },90^{\circ },135^{\circ }\}$. The function $\varepsilon_{ij}(\cdot )$ is a conditional function defined as

$$\varepsilon_{ij}(a,b)=\left\{ \begin{array}{cc} 1, & \text{ if }a=i\text{ and }b=j\\ 0, & \text{ otherwise } \end{array}\right..$$ (16)

Then, texture entropy and correlation are computed as:

$$G_{\text{Ent}}(\theta )=-\sum_{i=0}^{N-1}\sum_{j=0}^{N-1}G(i,j;\theta )\log G(i,j;\theta )$$ (17)

$$G_{\text{Cor}}(\theta )=\frac{\sum_{i=0}^{N-1}\sum_{j=0}^{N-1}(i-\mu_{i})(j-\mu_{j})G(i,j;\theta )}{\eta_{i}\eta_{j}}$$ (18)

where *N* is the number of gray levels. $\mu_{i}$ and $\mu_{j}$ are the mean values of the *i*th and *j*th gray levels, respectively. $\eta_{i}$ and $\eta_{j}$ represent the respective standard deviations of images.

The correlation value corresponding to the direction with the maximum texture entropy is selected as the detail feature.

#### 2.2.4 Cleanness level.

Cleanness reflects the degree of noise suppression in the image. A Gaussian filter *F*_*G*_ is applied to obtain a residual map:

$$D(x,y)=I(x,y)-(I*F_{G})(x,y)$$ (19)

A GGD is fitted to the residual map to extract the shape parameter *α* and variance $\sigma^{2}$. The entropy of the residual distribution is then computed as:

$$H_{D}=-\sum_{i=-255}^{255}p_{i}\log_{2}p_{i}$$ (20)

#### 2.2.5 Quality score regression.

The four attribute-specific feature vectors $f_{\text{RD}},f_{\text{GI}},f_{\text{DP}},f_{\text{CL}}$ are concatenated into a compact 10-dimensional feature vector *s*. This is input to a Support Vector Regression (SVR) model to produce a continuous quality score:

$$Q=\sum_{i=1}^{N}(y_{i}-y_{i}^{*})K(s_{i},s)+b$$ (21)

where K(·) denotes the Radial Basis Function (RBF) kernel, $y_{i}^{*}$ is the ground-truth quality score of the *i*-th training sample, and *s*_*i*_ is its corresponding feature vector.

## 3 Experimental results

Table 1 presents the perceptual quality assessment results of different filtering methods on three typical underwater sonar target categories (Ship, Human, and Aircraft), evaluated using UASIP (Unified Quality Assessment method for Sonar Imaging and Processing). As a unified standard for assessing underwater image quality, UASIP comprehensively considers structural preservation, noise suppression capability, and target saliency. It effectively measures how well a filtering method supports downstream perception tasks such as detection and recognition.

**Table 1:** UASIP scores (%) of different filtering methods on three sonar target categories.

Methods	Aircraft	Human	Ship
Original	58.3143	49.4454	52.8575
Median filtering	58.7307	53.4749	54.3685
Mean filtering	56.8897	51.4314	51.0448
Hybrid filtering	62.8821	54.5824	56.2959

Experimental results show that the hybrid filtering method achieved the highest UASIP scores across all target categories—62.8821%, 54.5824%, and 56.2959% for Aircraft, Human, and Ship, respectively—representing improvements of 4.57%, 5.14%, and 3.44% over the unprocessed original images. This hybrid method combines median filtering and bilateral filtering in series: the median filter effectively suppresses impulsive high-frequency noise, enhancing local robustness, while the bilateral filter further smooths the image guided by both spatial proximity and pixel similarity, all while preserving edge structures. The organic integration of these two filters leverages their respective strengths, achieving a sound balance between denoising and structural information preservation. This results in outstanding structural perception performance and strong cross-category adaptability.

In comparison, the median filtering method also yields relatively high UASIP scores for the Human and Ship categories, at 53.4749% and 54.3685%, respectively, and outperforms both mean filtering and the original images. This suggests that the median filter excels at preserving edge details, especially when dealing with images that have high local contrast or well-defined object contours. However, for the Aircraft category, its UASIP score is only slightly higher than that of the original image (58.7307% vs. 58.3143%), indicating limited enhancement in handling large-scale structures or regions with weak textures.

**Fig 1:**
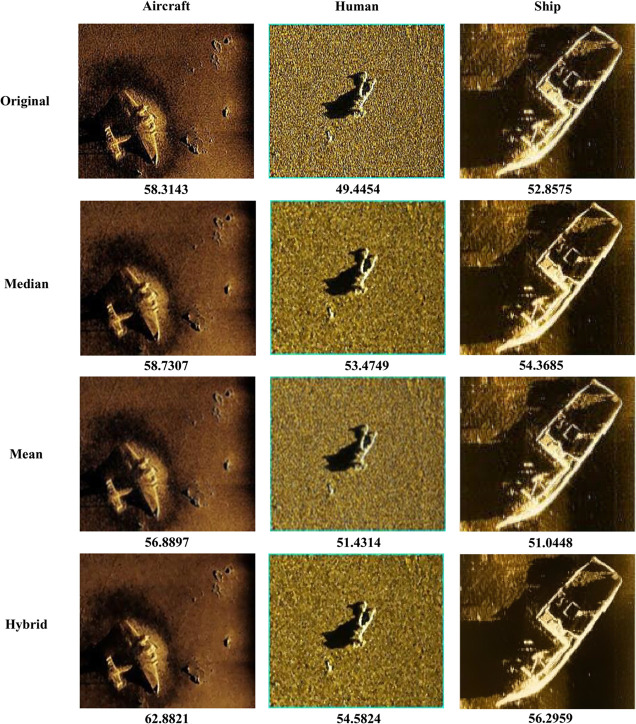
Filtering results comparison.

While the mean filtering method is computationally efficient and simple to implement, it performs poorly in terms of structural preservation. Its UASIP scores for the three target categories are 56.8897%, 51.4314%, and 51.0448%, consistently lower than those of the median and hybrid filtering methods. Mean filtering smooths the image by computing the arithmetic mean of neighboring pixels, which can reduce noise to some extent. However, it also tends to blur edges and weaken details, ultimately degrading structural perception. This makes it unsuitable for tasks that are highly sensitive to edge and texture information. In particular, the comparison results of the three different filters are shown in Fig 1.

## 4 Conclusion

In this paper, a two-stage hybrid filtering method is proposed to suppress noise and preserve edge information in the SAS images. Moreover, we adopt a no-reference quality assessment to evaluate image quality from perceptual and structural perspectives. From the experimental results and filtering effect, it is found that the proposed hybrid filtering method can achieve higher UASIP score than other filtering method, and the filtering effect of the proposed method is also better than the counterparts. The filtering method and quality assessment method are all important for SAS imaging, and we will further investigate these two parts to promote the SAS imaging technology in the future.

## References

[1] Zhu J, Yin T, Guo W, Zhang B, Zhou Z. An underwater target azimuth trajectory enhancement approach in BTR. Appl Acoust. 2025;230:110373

[2] Yin T, Guo W, Zhu J, Wu Y, Zhang B, Zhou Z. Underwater broadband target detection by filtering scanning azimuths based on features of subband peaks. IEEE Sensors J. 2025;25(8):13601–9. doi: 10.1109/jsen.2025.3546733

[3] Zhang X, Yang P, Feng X, Sun H. Efficient imaging method for multireceiver SAS. IET Radar Sonar & Navi. 2022;16(9):1470–83. doi: 10.1049/rsn2.12274

[4] Xu Z, Xu D, Lin L, Song L, Song D, Sun Y, et al. Integrated object detection and communication for synthetic aperture radar images. IEEE J Sel Top Appl Earth Observations Remote Sensing. 2025;18:294–307. doi: 10.1109/jstars.2024.3495023

[5] Liu M, Shang R, Liu K, Feng J, Wang C, Xu S, et al. Edge-enhanced cascaded MRF for SAR image segmentation. IEEE Trans Geosci Remote Sensing. 2025;63:1–11. doi: 10.1109/tgrs.2025.3561011

[6] Liu X, Li H, Zhu C. Joint contrast enhancement and exposure fusion for real-world image dehazing. IEEE Trans Multimedia. 2022;24:3934–46. doi: 10.1109/tmm.2021.3110483

[7] Zhang X, Yang P. Back projection algorithm for multi-receiver synthetic aperture sonar based on two interpolators. J Mar Sci Eng. 2022;10(6):718.

[8] Zhang X, Yang P, Wang Y, Shen W, Yang J, Ye K, et al. LBF-based CS algorithm for multireceiver SAS. IEEE Geosci Remote Sensing Lett. 2024;21:1–5. doi: 10.1109/lgrs.2024.3379423

[9] Zhang X. An efficient method for the simulation of multireceiver SAS raw signal. Multimed Tools Appl. 2023;83(13):37351–68. doi: 10.1007/s11042-023-16992-5

[10] Sun S, Xu Z, Cao X, Zheng J, Yang J, Jin N. A high-performance and lightweight maritime target detection algorithm. Remote Sensing. 2025;17(6):1012. doi: 10.3390/rs17061012

[11] Long H, Shen L, Wang Z, Chen J. Underwater forward-looking sonar images target detection via speckle reduction and scene prior. IEEE Trans Geosci Remote Sensing. 2023;61:1–13. doi: 10.1109/tgrs.2023.3248605

[12] Zhang X, Yang P, Sun M. Experiment results of a novel sub-bottom profiler using synthetic aperture technique. Curr Sci. 2022;122(4).

[13] Zhang X, Yang P, Cao D. Synthetic aperture image enhancement with near-coinciding nonuniform sampling case. Comput Electr Eng. 2024;120:109818.

[14] Si J, Zhou T, Yu X, Du W, Xu S. An unsupervised method for detecting and segmenting shadow areas of sunken targets in sonar images. IEEE Trans Instrum Meas. 2025;74:1–15. doi: 10.1109/tim.2025.3527611

[15] Xu Z, Tang B, Ai W, Xie Z, Zhu J. Radar transceiver design for extended targets based on optimal linear detector. IEEE Trans Aerosp Electron Syst. 2025;61(3):6070–82. doi: 10.1109/taes.2024.3524951

[16] Zhang X, Yang P, Sun H. Frequency-domain multireceiver synthetic aperture sonar imagery with Chebyshev polynomials. Electron Lett. 2022;58(25):995–8.

[17] Gerg ID, Cotner CF. A perceptual metric prior on deep latent space improves out-of-distribution synthetic aperture sonar image classification. In: IGARSS 2023 - 2023 IEEE International Geoscience and Remote Sensing Symposium. 2023. p. 6576–9. 10.1109/igarss52108.2023.10283358

[18] Su J, Xu D, Qiu L, Xu Z, Lin L, Zheng J. A high-accuracy underwater object detection algorithm for synthetic aperture sonar images. Remote Sensing. 2025;17(13):2112. doi: 10.3390/rs17132112

[19] Wu H, Zhou J. IID-Net: image inpainting detection network via neural architecture search and attention. IEEE Trans Circuits Syst Video Technol. 2022;32(3):1172–85. doi: 10.1109/tcsvt.2021.3075039

[20] Xu Z, Tang B, Ai W, Zhu J. Relative entropy based jamming signal design against radar target detection. IEEE Trans Signal Process. 2025;73:1200–15. doi: 10.1109/tsp.2025.3544305

[21] Mittal S, Srivastava S, Jayanth JP. A survey of deep learning techniques for underwater image classification. IEEE Trans Neural Netw Learn Syst. 2023;34(10):6968–82. doi: 10.1109/TNNLS.2022.3143887 35104229

[22] Zhu J, Song Y, Jiang N, Xie Z, Fan C, Huang X. Enhanced doppler resolution and sidelobe suppression performance for golay complementary waveforms. Remote Sensing. 2023;15(9):2452. doi: 10.3390/rs15092452

[23] Zhang Y, Jiang G, Cai Z, Zhou Y. Bipartite graph-based projected clustering with local region guidance for hyperspectral imagery. IEEE Trans Multimedia. 2024;26:9551–63. doi: 10.1109/tmm.2024.3394975

[24] You X, Crebbin G. Robust adaptive estimator for filtering noise in images. IEEE Trans Image Process. 1995;4(5):693–9. doi: 10.1109/83.382505 18290020

[25] Guo S, Delbruck T. Low cost and latency event camera background activity denoising. IEEE Trans Pattern Anal Mach Intell. 2023;45(1):785–95. doi: 10.1109/TPAMI.2022.3152999 35196224

[26] Zhang X, Yang P, Wang Y, Shen W, Yang J, Wang J, et al. A novel multireceiver SAS RD processor. IEEE Trans Geosci Remote Sensing. 2024;62:1–11. doi: 10.1109/tgrs.2024.3362886

[27] Zhao W, Yan J, Jin D, Ling J. C-HRNet: high resolution network based on contexts for single-frame phase unwrapping. IEEE Photonics Journal. 2024;16.

[28] Cao N, Liu Y. High-noise grayscale image denoising using an improved median filter for the adaptive selection of a threshold. Appl Sci. 2024;14(2):635.

[29] Wu Y, Hui C, Liao R, Liu S, Zhao D. Image compressive sensing with adaptive sampling by median filtering. In: ICASSP 2025 -2025 IEEE Int Conf Acoust Speech Signal Process (ICASSP). IEEE; 2025; p. 1–5.

[30] Gerg ID, Monga V. Structural prior driven regularized deep learning for sonar image classification. IEEE Trans Geosci Remote Sensing. 2022;60:1–16. doi: 10.1109/tgrs.2020.3045649

[31] Jian J, Liu L, Zhang Y, Xu K, Yang J. Optical remote sensing ship recognition and classification based on improved YOLOv5. Remote Sensing. 2023;15(17):4319. doi: 10.3390/rs15174319

[32] Algburi RNA, Aljibori HSS, Al-Huda Z, Gu YH, Al-antari MA. Advanced fault diagnosis in industrial robots through hierarchical hyper-laplacian priors and singular spectrum analysis. Complex Intell Syst. 2025;11(6). doi: 10.1007/s40747-025-01915-8

[33] Algburi RNA, Gao H, Al-Huda Z. Implementation of singular spectrum analysis in industrial robot to detect weak position fluctuations. Fluct Noise Lett. 2020;20(03):2150010. doi: 10.1142/s0219477521500103

[34] Sun XX, Qu W. Comparison between mean filter and median filter algorithm in image denoising field. Appl Mech Mater. 2014;644:4112–6.

[35] Shah A, Bangash JI, Khan AW, Ahmed I, Khan A, Khan A, et al. Comparative analysis of median filter and its variants for removal of impulse noise from gray scale images. Journal of King Saud University - Computer and Information Sciences. 2022;34(3):505–19. doi: 10.1016/j.jksuci.2020.03.007

[36] Vishwakarma A. Denoising and inpainting of sonar images using convolutional sparse representation. IEEE Trans Instrum Meas. 2023;72:1–9. doi: 10.1109/tim.2023.324652737323850

[37] Zheng J, Zhao S, Xu Z, Zhang L, Liu J. Anchor boxes adaptive optimization algorithm for maritime object detection in video surveillance. Front Mar Sci. 2023;10:1290931.

[38] Zhang X, Yang P, Sun H. An omega-k algorithm for multireceiver synthetic aperture sonar. Electron Lett. 2023;59(13):e12859.

[39] Kong X, Yang C, Cao S, Li C, Peng Z. Infrared small target detection via nonconvex tensor fibered rank approximation. IEEE Trans Geosci Remote Sensing. 2022;60:1–21. doi: 10.1109/tgrs.2021.3068465

[40] Zhang X, Yang P. An improved imaging algorithm for multi-receiver SAS system with wide-bandwidth signal. Remote Sensing. 2021;13(24):5008. doi: 10.3390/rs13245008

[41] Tiwari V, Prakash S. Enhancement of farming using IoT based unmanned aerial vehicles. In: 2024 2nd International Conference on Disruptive Technologies (ICDT), 2024. p. 941–8. 10.1109/icdt61202.2024.10489272

[42] La Fata A, Moser G, Procopio R, Bernardi M, Fiori E. A Gaussian process regression method to nowcast cloud-to-ground lightning from remote sensing and numerical weather modeling data. IEEE J Sel Top Appl Earth Observations Remote Sensing. 2025;18:1963–81. doi: 10.1109/jstars.2024.3501976

[43] Tong X, Su S, Wu P, Guo R, Wei J, Zuo Z, et al. MSAFFNet: a multiscale label-supervised attention feature fusion network for infrared small target detection. IEEE Trans Geosci Remote Sensing. 2023;61:1–16. doi: 10.1109/tgrs.2023.3279253

[44] Alika şifo ğlu T, Kartal B, Ko ç A. Wiener filtering in joint time-vertex fractional fourier domains. IEEE Signal Process Lett. 2024;31:1319–23. doi: 10.1109/lsp.2024.3396664

[45] Zhang W, Huang B, Chen S, Xu X, Wu W, Liu Q. Low-rank angular prior guided multi-diffusion model for few-shot low-dose CT reconstruction. IEEE Trans Comput Imaging. 2024;10:1763–74.

[46] Guo S, Shi C, Wang L, Jin J, Lu Y. EBF: an event-based bilateral filter for effective neuromorphic vision sensor denoising. IEEE Transactions on Circuits and Systems for Video Technology. 2025:1.

[47] Wu Z, Zhong X, Lyv T, Wang D, Chen R, Yan X, et al. Deep dual-domain united guiding learning with global–local transformer-convolution U-Net for LDCT reconstruction. IEEE Trans Instrum Meas. 2023;72:1–15. doi: 10.1109/tim.2023.332920037323850

[48] Mishiba K. Fast guided median filter. IEEE Trans Image Process. 2023;32:737–49. doi: 10.1109/TIP.2022.3232916 37018332

[49] Schaffenroth S, Schmidt H-P, Kolpin A. Mitigation of coloured impulsive noise in OFDM receiver. In: 2021 IEEE International Symposium on Power Line Communications and its Applications (ISPLC). 2021. p. 43–8. 10.1109/isplc52837.2021.9628650

[50] Cai B, Chen W, Zhang J, Junejo NUR, Zhao T. Unified no-reference quality assessment for sonar imaging and processing. IEEE Transactions on Geoscience and Remote Sensing. 2024;63.

[51] Zhu J, Xie Z, Jiang N, Song Y, Han S, Liu W, et al. Delay-doppler map shaping through oversampled complementary sets for high-speed target detection. Remote Sensing. 2024;16(16):2898. doi: 10.3390/rs16162898

[52] Zhang X, Dai X, Yang B. Fast imaging algorithm for the multiple receiver synthetic aperture sonars. IET Radar Sonar & Navi. 2018;12(11):1276–84. doi: 10.1049/iet-rsn.2018.5040

